# Exacerbation of delirium and epileptic seizures in an older man with idiopathic Parkinson’s disease due to multiple prescriptions: a case report

**DOI:** 10.3389/fmed.2024.1415988

**Published:** 2024-07-18

**Authors:** Takuya Yamaguchi, Akinobu Aihara, Shigeto Mashiko, Emiko Kurosawa, Tomoya Oizumi, Toshihiro Yamagata, Aiko Ishiki, Juri Ueda, Yuko Fujikawa, Atsuhiro Kanno, Kazuhiro Sumitomo, Takahiro Ohara, Katsutoshi Furukawa

**Affiliations:** Division of Geriatric and Community Medicine, Faculty of Medicine, Tohoku Medical and Pharmaceutical University, Sendai, Japan

**Keywords:** Parkinson’s disease, polypharmacy, delirium, Parkinson’s disease psychosis, renal dysfunction

## Abstract

**Introduction:**

Parkinson’s disease (PD) is a disorder characterized by motor symptoms, such as rigidity, akinesia, and resting tremor, as well as non-motor symptoms, including psychiatric manifestations and autonomic failure. The prevalence of PD increases with age, and the condition is more common in men than in women. Conversely, polypharmacy has emerged as a paramount medical concern, especially among older patients, correlating with medicines’ adverse effects, interactions between medicines, frequent admissions to the hospital, and a high risk of morbidity and mortality.

**Case description:**

We encountered an older male patient with idiopathic PD and mild renal dysfunction. Originally prescribed 14 types of medicines, including anti-PD drugs, the patient developed delirium and epileptic seizures during hospitalization. After reducing the number of medications, including amantadine, the symptoms significantly improved. This clinical course suggests that polypharmacy, in addition to PD itself, poses a significant risk of delirium and epileptic seizures, even in patients with mild renal dysfunction.

**Conclusion:**

This report is indicative of the risk of polypharmacy and highlights the importance of citing drug interactions for a correct diagnosis in patients presenting with complex symptoms.

## 1 Introduction

Parkinson’s disease (PD) is characterized by the degeneration of dopaminergic neurons in the substantia Nigra. The condition causes motor symptoms such as rigidity, immobility, and resting tremors, as well as non-motor symptoms, including psychiatric and autonomic symptoms. The prevalence of PD increases with age, reaching a peak at 85–89 years of age, and is more common in men than in women. Although PD is idiopathic in most patients, genetic and environmental factors are also associated with this disorder (1). The Braak hypothesis is the most widely cited explanation for the neurological progression of PD (2). This hypothesis indicates that PD originates in the dorsal vagal nucleus and olfactory bulb, and progresses to the cerebral cortex, resulting in cognitive deficits and hallucinations, particularly in advanced PD. Therefore, dopamine replacement is now believed to be the most commonly used treatment for PD (1, 3).

In addition to the development of several novel anti-PD medicines, polypharmacy is one of the most crucial medical issues, especially among older individuals (4, 5). Furthermore, both PD and its pharmacological treatment frequently induce psychosis and delirium. Many researchers have extensively discussed the pathogenesis of delirium and epileptic seizures in idiopathic PD. Psychosis rarely occurs, and delusions are less frequent than hallucinations in patients with untreated idiopathic PD (5). Furthermore, hallucinations in idiopathic PD have been identified as multifactorial entities dependent on extrinsic and intrinsic mechanisms, including genetic, anatomical, neurotransmission-mediated, and environmental factors (6). The pathomechanism of hallucinations and delusions in dementia with Lewy bodies is considered identical to that of levodopa-induced psychosis (7). Starr reported that alteration of dopamine neurotransmission is one of the key contributors to the pathogenesis of epileptic seizures. In addition, the proconvulsant properties of selective D1 agonists indicate that dopamine lowers the seizure threshold (8).

We encountered a patient with idiopathic PD (Hoehn and Yahr stage: 3 and Movement Disorder Society Unified Parkinson’s Disease Rating Scale: 108), who was originally prescribed dopaminergic medicines. The interaction of these medications with multiple agents may have triggered delirium and epileptic seizures after admission. As we consider this case suggestive, we report it along with a literature review.

## 2 Case presentation

A man in his 70s came to our hospital complaining of weight loss, anorexia, and dysphagia. His medical history included hypertension and dyslipidemia, in addition to PD. He had mild dementia because his mimimental state examination score was 20. He had been taking medication for PD for 12 years, and his weight loss commenced in the last year of admission, with a loss of 10 kg in the last three months. In October of the same year, fatigue, dizziness, dysuria, and severe constipation developed along with a marked decrease in food intake. A few days before visiting our hospital, he visited a nearby hospital and underwent esophagogastroduodenoscopy and cranial magnetic resonance imaging; however, the results were negative. Blood tests revealed mild renal dysfunction (estimated glomerular filtration rate: 36.9 mL/min/1.73 m^2^), mild inflammatory response (C-reactive protein: 1.24 mg/dL), and anemia (hemoglobin: 8.2 g/dL). He was admitted to our hospital for further examination and tests for weight loss, anorexia, and anemia were performed. Upon admission, a general physical examination revealed no significant abnormalities. However, a neurological examination revealed resting tremors, bladder and bowel incontinence, and orthostatic hypotension. In contrast, the mask-like facial appearance and muscle rigidity were very mild, even though his swallowing function significantly declined. He developed epileptic seizures and delirium on the morning of the 3rd day after amantadine and droxidopa was initiated for orthostatic hypotension. The total number of his medicines was 14 then (Table 1). His epileptic seizures were considered as tonic–clonic, although he did not have status epileptics throughout the clinical course. The patient’s delirium was considered as a mixed type. Confusion Assessment Method (CAM) short form was applied and his condition met the CAM short form diagnostic criteria (9). The brain magnetic resonance imaging only showed mild cerebral atrophy, and the patient did not have any history of cerebrovascular diseases or seizures in the past. The electroencephalogram showed repetitive sharp waves and spike-and-wave complexes in the left frontal and temporal lobes, but any significant electrolytes imbalances were not observed including serum magnesium (2.3 mg/dL) and no magnesium supplement had been taken. Although the patient showed a mild loss of appetite, he did not have any infections including pneumonia or urinary tract infection. Because these results suggested intoxication from dopaminergic medicines, amantadine and droxidopa were discontinued. After discontinuation of amantadine and droxidopa, we increased the doses of levodopa (600 mg/day) and entacapone (600 mg/day) to 1,000 mg/day for each medicine. After these adjustments, his Parkinsonism was well controlled and no adverse reactions such as epileptic seizure, delusion or delirium were observed for half a year. Furthermore, his anorexia was improved but urinary symptoms or constipation was not changed drastically. In addition, he underwent a total colonoscopy for further examination, but no findings explaining the weight loss or any other issues were identified. Based on the patient’s clinical course, we concluded that his multiple manifestations were attributable to polypharmacy, including the use of anti-PD medicines.

**Table 1:** Prescribed medicines.

Drug	Dosage
Amantadine	200 mg/day
Droxidopa	100 mg/day
Istradefylline	40 mg/day
Rasagiline	1 mg/day
Risperidone	1 mg/day
Fenofibrate	160 mg/day
Amlodipine	5 mg/day
Lubiprostone	24 mg/day
Magnesium oxide	1.32 g/day
Mosapride	5 mg/day
Elobixibat	10 mg/day
Sennoside	24 mg/day
Vonoprazan	20 mg/day
Stalevo L100	6 tablets/day (Levodopa 600 mg/day, Entakapon 600 mg/day)

## 3 Discussion

The prevalence of psychosis in cross-sectional studies of PD is 13–60% depending on the selected diagnostic criteria and specific population. Moreover, the lifetime prevalence of PD is 47–60% (1–3). PD psychosis affects the quality of life and contributes to physical disability, in addition to increasing caregiver burden and distress (10). The prevalence of psychosis in untreated *de novo* PD was reported to be 3% (11). Additionally, dopamine replacement therapy may be associated with an increased frequency of psychiatric symptoms (12). Psychiatric symptoms, encompassing minor phenomena, visual hallucinations, non-visual hallucinations, delusions, and visual illusions, are particularly common and occur frequently at night in dim lighting conditions. Several differences exist between hallucinations in primary psychiatric disorders and psychosis in PD. For instance, hallucinations in schizophrenia are more frequent and auditory than those in PD psychosis (10). Although the pathophysiology of PD psychosis is intricate and not fully elucidated, most dopaminergic medicines for PD have been observed to induce psychotic symptoms. Consequently, dopamine receptor antagonists are often employed in the treatment of psychosis. Thus, chronic dopaminergic treatment may result in mesolimbic dopamine receptor hypersensitivity, which may contribute to the development of psychosis (13). PD psychosis is also associated with rapid eye movement sleep behavior disorders and sleep abnormalities (14, 15).

Many neurotransmitters, including dopamine, are involved in the development of epilepsy, with dopamine playing an important role in its regulation. The modulation of seizures also depends on the dopamine receptor subtypes and brain regions in which dopamine receptors are activated. Various animal and human studies have demonstrated that D1-like and D2-like receptor signaling exert opposing effects on limbic epileptogenesis. In general, signaling through D1-like receptors promotes epileptogenesis by lowering the threshold and increasing the severity of epileptic seizures (16, 17). In humans, high D1 expression is observed in the neocortex of patients with temporal lobe epilepsy, and D1 binding is positively correlated with the duration of epilepsy (18). In contrast, D2-like receptor signaling is generally considered to possess antiepileptic properties, and antagonizing D2-like receptor signaling lowers the seizure threshold (19, 20). Importantly Gruntz et al. reported that PD is associated with an elevated risk of epileptic seizures, indicating that the adjusted odds ratio of epileptic seizures was 1.68 (95% confidence interval = 1.43–1.98) in patients with PD compared to that in individuals without PD (21).

Today, polypharmacy, which is usually defined as the regular use of five or more drugs, is a very important medical issue, due to its association with drug-related problems, frequent hospital admissions, a high risk of morbidity and mortality, and a high economic burden, including medical bills (22, 23). In an observational study involving Japanese individuals, approximately 75% of participants consumed five or more drugs. The study demonstrated that the number of medicines was associated with the risk of renal failure, cardiovascular events, and mortality (24). According to a study by Naghnaghia et al., older adults with excessive polypharmacy (10 or more agents) were 2.7 times more likely to have renal dysfunction compared to those consuming fewer than five agents (25).

Amantadine acts on the dopaminergic neurons and is used to treat PD (26). In Japan, amantadine is approved and covered by insurance for this purpose owing to its effectiveness in ameliorating reduced spontaneity resulting from cerebral infarction. However, as amantadine is excreted primarily via renal clearance, the half-life of the drug tends to be prolonged in patients with renal dysfunction (27). Although some reports have highlighted amantadine intoxication in patients with advanced renal failure (28, 29), the number of reported cases of amantadine intoxication in patients with mild to moderate renal dysfunction is lower than that in patients with severe renal failure (27–29). Amantadine toxicity primarily affects the central nervous system (CNS) and manifests as hallucinations, confusion, and nightmares. Other CNS manifestations including insomnia, fatigue, drowsiness, acute psychosis, coma, and seizures have also been reported (28, 29). Although the interaction between droxidopa and amantadine has rarely been reported, the balance of neurotransmitters and their interactions should be associated with the pathophysiology. It was reported that amantadine inhibits NMDA receptor and this action has a beneficial effect on PD. It can also disrupt normal brain signaling, which can contribute to confusion and delirium. NMDA receptor inhibition may lower the seizure threshold in some individuals. Amantadine may also have mild anticholinergic properties, which can interfere with the balance of acetylcholine in the brain. This can contribute to cognitive decline and confusion, potentially leading to delirium (28–29). Therefore, regardless of renal function, administering amantadine or other anti-PD medications should be carefully considered, especially in a context of geriatric patients who already experience the issue of polypharmacy.

This patient, who was originally consuming 14 medicines (Table 1), including six anti-PD medicines, presented with seizures and delirium after admission to the hospital. Based on the clinical course, the patient’s symptoms were speculated to have occurred due to elevated blood amantadine levels triggered by droxidopa. Considering the patient’s moderately impaired renal function, it is improbable that renal function alone caused amantadine intoxication, suggesting that polypharmacy also contributed.

Polypharmacy is a serious issue in older adults, with overmedication leading to many unfavorable events. As multiple medicines regulate the balance of several neurotransmitters in PD, adding one agent could disrupt the homeostasis of the neuronal network. It may be useful to promote deprescribing in the everyday clinical practice rather than adding a medication for a new symptom or an adverse reaction. Considering the aforementioned information, treating patients with the minimum amount of medication whenever feasible is important. To achieve this goal, drug adjustments and prescriptions are recommended. In addition, patients with PD consuming multiple agents are at risk of developing amantadine intoxication due to drug interactions, even if the renal dysfunction is mild to moderate. This report emphasizes the risk of polypharmacy and suggests the importance of citing amantadine intoxication as a differential diagnosis in patients with PD and mild to moderate renal dysfunction who present with symptoms of seizures and/or delirium.

## Data availability statement

The original contributions presented in the study are included in the article/supplementary material, further inquiries can be directed to the corresponding author.

## Ethics statement

The studies involving humans were approved by Tohoku Medical and Pharmaceutical University. The studies were conducted in accordance with the local legislation and institutional requirements. The participants provided their written informed consent to participate in this study. Written informed consent was obtained from the individual(s) for the publication of any potentially identifiable images or data included in this article.

## Author contributions

TaY: Writing – original draft, Data curation. AA: Data curation, Writing – review & editing. SM: Data curation, Writing – review & editing. EK: Data curation, Writing – review & editing. ToO: Data curation, Writing – review & editing. ToY: Writing – review & editing. AI: Data curation, Writing – original draft. JU: Data curation, Writing – review & editing. YF: Writing – review & editing. AK: Writing – review & editing. KS: Writing – review & editing. TaO: Writing – review & editing. KF: Writing – original draft.
